# Toll‐7 promotes tumour growth and invasion in *Drosophila*

**DOI:** 10.1111/cpr.13188

**Published:** 2022-01-20

**Authors:** Xiang Ding, Zhuojie Li, Gufa Lin, Wenzhe Li, Lei Xue

**Affiliations:** ^1^ Institute of Intervention Vessel Shanghai 10th People's Hospital Shanghai Key Laboratory of Signaling and Disease Research School of Life Science and Technology Tongji University Shanghai China; ^2^ Key Laboratory of Spine and Spinal Cord Injury Repair and Regeneration of Ministry of Education Orthopaedic Department of Tongji Hospital School of Life Sciences and Technology Tongji University Shanghai China; ^3^ Zhuhai Precision Medical Center Zhuhai People's Hospital Zhuhai Hospital Affiliated with Jinan University Zhuhai China

**Keywords:** *Drosophila*, EGFR, hippo, JNK, Toll‐7, tumorigenesis

## Abstract

**Objectives:**

*Drosophila melanogaster* has become an excellent model organism to explore the genetic mechanisms underlying tumour progression. Here, by using well‐established *Drosophila* tumour models, we identified Toll‐7 as a novel regulator of tumour growth and invasion.

**Materials and methods:**

Transgenic flies and genetic epistasis analysis were used. All flies were raised on a standard cornmeal and agar medium at 25°C unless otherwise indicated. Immunostaining and RT‐qPCR were performed by standard procedures. Images were taken by OLYMPUS BX51 microscope and Zeiss LSM 880 confocal microscope. Adobe Photoshop 2020 and Zeiss Zen were used to analyse the images. All results were presented in Scatter plots or Column bar graphs created by GraphPad Prism 8.0.

**Results:**

Loss of *Toll*‐*7* suppresses Ras^V12^/*lgl* ^−/−^‐induced tumour growth and invasion, as well as cell polarity disruption‐induced invasive cell migration, whereas expression of a constitutively active allele of Toll‐7 is sufficient to promote tumorous growth and cell migration. In addition, the Egr‐JNK signalling is necessary and sufficient for Toll‐7‐induced invasive cell migration. Mechanistically, Toll‐7 facilitates the endocytosis of Egr, which is known to activate JNK in the early endosomes. Moreover, Toll‐7 activates the EGFR‐Ras signalling, which cooperates with the Egr‐JNK signalling to promote Yki‐mediated cell proliferation and tissue overgrowth. Finally, Toll‐7 is necessary and sufficient for the proper maintenance of EGFR protein level.

**Conclusions:**

Our findings characterized Toll‐7 as a proto‐oncogene that promotes tumour growth and invasion in *Drosophila*, which shed light on the pro‐tumour function of mammalian Toll‐like receptors (TLRs).

## Graphical abstract

In this study, we identified Toll‐7 as a proto‐oncogene that promotes tumour growth and invasion through Egr‐JNK and EGFR‐Ras signalling. Mechanistically, Toll‐7 facilitates the endocytosis of Egr, which is known to activate JNK in the early endosomes. Toll‐7 promotes EGFR expression post‐transcriptionally to activate ERK. Our results provide in‐vivo evidence and underlying genetic mechanism for the role of Toll‐7 in promoting tumorigenesis.

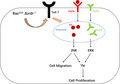

## 1 INTRODUCTION

Cancer has become a major health problem and is expected to be the first leading cause of death worldwide.^1^ Cancer progression requires interaction between multiple genes and crosstalk of signalling pathways.^2^, ^3^, ^4^, ^5^ Despite the characterization of many proto‐oncogenes and tumour suppressors,^6^, ^7^, ^8^ additional factors involved in this process remain to be unveiled. Due to its low genetic redundancy, *Drosophila* has become an excellent in‐vivo system to identify novel tumour‐related genes.^9^, ^10^ Thus far, several tumour models have been established and widely utilized in fly. For instance, cell polarity genes such as *scribble* (*scrib*) and *lethal giant larvae* (*lgl*) encode tumour suppressors,^3^, ^11^, ^12^ whose mutation cooperates with oncogenic Ras to promote tumorous growth and metastasis in the larval eye discs.^13^, ^14^, ^15^ In addition, depletion of tumour suppressor gene *csk* or cell polarity genes in the A/P boundary region of larval wing discs induces invasive cell migration.^12^, ^16^ Further researches indicate that the c‐Jun N‐terminal kinase (JNK) signalling plays a critical role in these tumour models.^17^, ^18^

The JNK pathway, which is evolutionarily conserved from fly to human, plays pivotal roles in a wide range of cellular processes including cell death, proliferation, differentiation and migration.^19^, ^20^ In *Drosophila*, JNK signalling is activated by Eiger (Egr), the orthologue of tumour necrosis factor (TNF).^21^, ^22^ Egr binds to its receptor Grindelwald and recruits the adaptor protein tumour necrosis factor receptor‐associated factor 2 (dTRAF2),^23^, ^24^ which, in turn, activates the JNK cascade including JNKK Kinase dTAK1, JNK Kinase Hemipterous (Hep) and JNK Basket (Bsk).^25^, ^26^ Activation of JNK leads to phosphorylation and activation of the transcription factors Jun and Fos, which translocate to the nucleus and regulate target gene expression.^27^, ^28^

The Toll signal pathway was first identified as a regulator of dorsal‐ventral axis formation in *Drosophila* embryos^29^ and was subsequently found to play an important role in the innate immune response.^30^ Besides the trans‐membrane receptor Toll, other components of the canonical Toll pathway include Spätzle, Myd88, Tube, Pelle, Cactus, Dorsal and Dif.^31^, ^32^ Upon binding to the ligand Spätzle, Toll recruits Tube and Pelle through the adaptor protein Myd88. Activated Pelle phosphorylates Cactus and triggers its degradation, which releases the transcription factors Dorsal and Dif, allows their entry into the nucleus to activate the expression of target genes.^33^, ^34^, ^35^

The Toll signal pathway is highly conserved from insect to mammal, with the mammalian counterparts of Toll being named Toll‐like receptors (TLRs).^36^ To date, nine Toll family members have been identified in *Drosophila*. While the roles of Toll in embryonic patterning and innate immunity have been well‐documented, recent studies suggest Toll signalling is also involved in other biological processes including cell death, wound healing and cell competition.^35^, ^37^, ^38^, ^39^ Furthermore, crosstalk between Toll signalling and other pathways, for example JNK or Hippo, has been reported to regulate cell death or immunity.^40^, ^41^ Yet, the functions of other Toll family members remain largely unexplored.

In this study, we identified Toll‐7 as a proto‐oncogene that promotes tumour growth and invasion by activating both Egr‐JNK and EGFR‐Ras signalling. The Egr‐JNK signalling is necessary and sufficient to trigger invasive cell migration, while the EGFR‐Ras signalling cooperates with the Egr‐JNK signalling to promote Yki‐mediated cell proliferation and tissue overgrowth. Mechanistically, Toll‐7 facilitates the endocytosis of Egr, which is known to activate JNK in the early endosomes. In addition, Toll‐7 promotes EGFR expression post‐transcriptionally. Therefore, these data provide in‐vivo evidence and underlying genetic mechanism for the role of Toll‐7 in promoting tumour growth and invasion, which shed light on the pro‐tumour function of mammalian TLRs.

## 2 MATERIALS AND METHODS

### 2.1 Fly strains

All flies were raised on a standard cornmeal and agar medium at 25°C unless otherwise indicated. Fluorescently labelled invasive tumours were dissected in 3rd instar larval eye discs using the following strains: *yw*, *ey*‐FLP1; *tub*‐Gal80, FRT40A; *act*>y^+^>Gal4, *UAS*‐GFP (40A tester), *lgl* ^4^, FRT40A, *UAS*‐Ras^V12^. Additional fly stocks used in this study: *ptc*‐Gal4, *GMR*‐Gal4^s^, *GMR*‐Gal4, *UAS*‐GFP, *UAS*‐*GFP*‐*IR*, *hh*‐Gal4, *UAS*‐LacZ, *UAS*‐Bsk^DN^, *UAS*‐Puc, *UAS*‐Egr^W^, *UAS*‐Egr^Regg^, *act*‐Gal4, *tub*‐Gal80^ts^, *UAS*‐*scrib*‐*RNAi*, *diap1*‐LacZ, *wg*‐LacZ, *ex*‐LacZ, *ban*‐LacZ were previously described.^24^, ^28^, ^59^, ^70^ *UAS*‐Myc‐Egr‐HA was obtained from Xue lab. *UAS*‐Toll‐7^CY^ was a kind gift from Dr. A. Hidalgo, *aos*‐LacZ (BL2513) was a kind gift from Dr. Jian Zhu. *UAS*‐*Toll*‐*7*‐*RNAi* (30488), *UAS*‐Rab5‐GFP (43336), *UAS*‐*EGFR*‐*RNAi* (25781), *UAS*‐EGFR (5368) were obtained from the Bloomington stock center, *UAS*‐*Toll*‐*7*‐*RNAi* (39176), *UAS*‐*yki*‐*RNAi* (40497), *UAS*‐*egr*‐*RNAi* (45253) were obtained from the VDRC stock center.

### 2.2 Immunostaining

Antibody staining of imaginal discs was performed by standard procedures. Primary antibodies included mouse anti‐MMP1 (1:200; DSHB 3A6B4), rabbit anti‐Cleaved Dcp‐1 (1:100; CST 9578), Phalloidin 555 (1:200; CST 8953S), rat anti‐DE‐cadherin (1:100; DSHB DCAD2‐c), mouse anti‐β‐integrin (1:100; DSHB C‐F.6G11‐c), rabbit anti‐phospho‐Histone H3 (1:400; CST 9701), rabbit anti‐phospho‐JNK (1:200; Calbiochem #559309), mouse anti‐DLG (1:100; DSHB 4F3), rabbit anti‐Egr (1:100; gift from Dr. M. Miura), rabbit anti‐Rab5 (1:500; abcam ab31261), mouse anti‐Myc‐Tag (1:100 CST 2276), mouse anti‐β‐Gal (1:500; DSHB 40‐1a), mouse anti‐Wg (1:100; DSHB 4D4) and mouse anti‐EGFR (1:100; Sigma‐Aldrich E2906). Second antibodies included goat anti‐rabbit‐Cy3 (1:1000; Life technologies A10520), goat anti‐mouse‐Cy3 (1:1000; Life technologies A10521), goat anti‐mouse‐Cy5 (1:1000; Life technologies A10524) and goat anti‐rat‐Cy3 (1:1000; Life technologies A10522).

### 2.3 RT‐qPCR

To assess the knockdown efficiencies of *Toll*‐*7 RNAi* lines, *act*‐Gal4; *tub*‐Gal80^ts^ driver was used. Animals were raised at 25°C for 2 day, and then shifted to 29°C for 3 day before dissecting the larvae.

Primers used were as follows:

rp49‐FP: TACAGGCCCAAGATCGTGAA.

rp49‐RP: TCTCCTTGCGCTTCTTGGA.

Toll‐7‐FP: ATCCATCGCAACCCAGTGG.

Toll‐7‐RP: GCTGTCGCTCAATGAGACG.

### 2.4 Statistical analysis

Adobe Photoshop 2020 was used to measure the tumour size and quantified as relative volume to that of wild‐type. Adobe Photoshop 2020 was also used to count the migrating cell number. All results were presented in Scatter plots or Column bar graphs created by GraphPad Prism 8.0. A combination of One‐way ANOVA with Bonferroni's multiple comparison test and the *T*‐test were used to compute *p*‐values, *p*‐value < 0.05 was considered as significant. n.s means not significant. *, **, **** and **** represent *p*‐value less than 0.05, 0.01, 0.001 and 0.0001, respectively.

## 3 RESULTS

### 3.1 Loss of *Toll*‐*7* suppresses Ras^V12^/*lgl* ^−/−^ induced tumour growth and invasion

**FIGURE 1:**
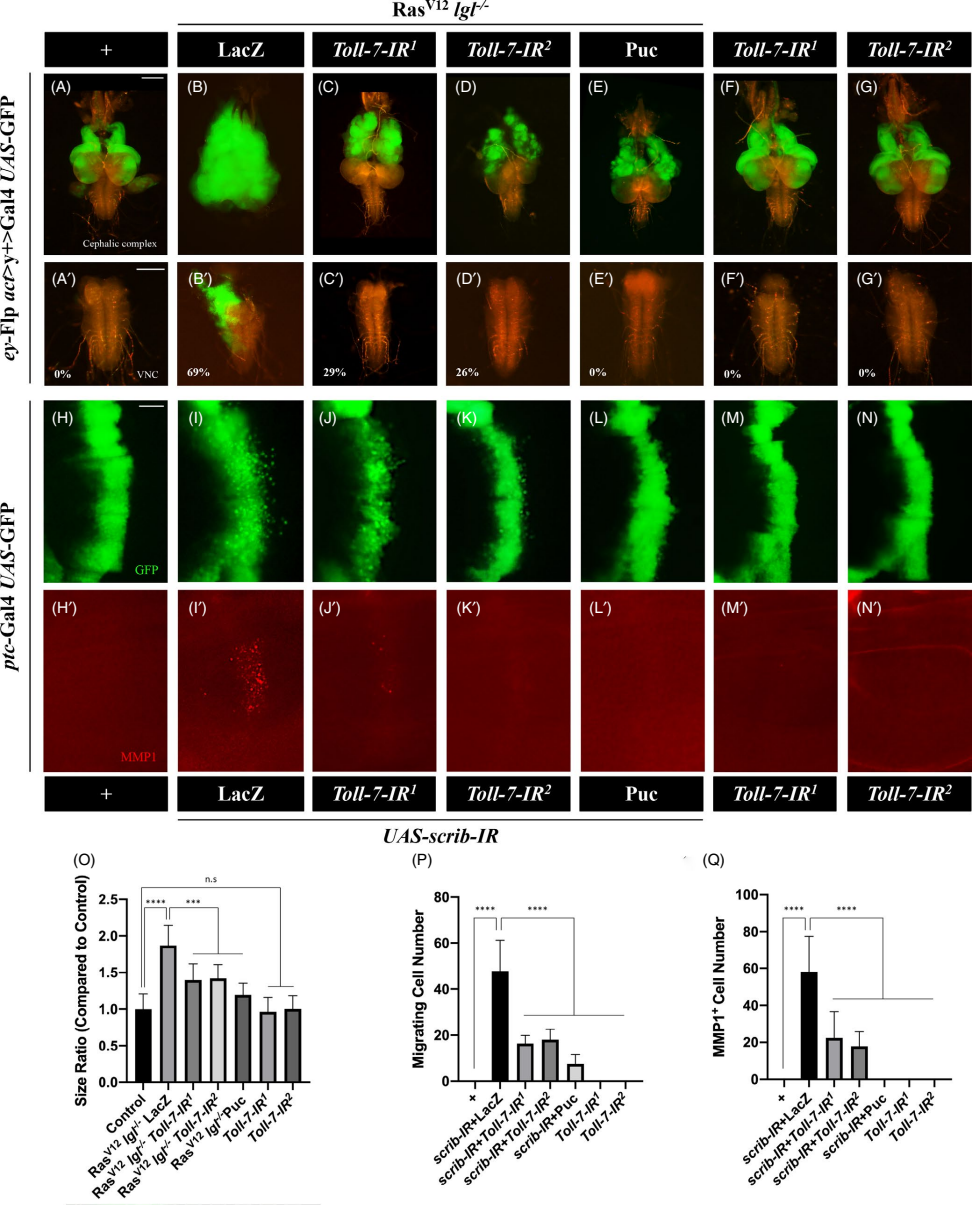
Loss of *Toll*‐*7* suppresses tumor growth and invasion in *Drosophila*. Fluorescent images of *Drosophila* larval cephalic complexes (A–G) and Ventral Nerve Cords (VNC, A’–G’) are shown. GFP‐labelled MARCM clones were created in the eye‐antennal discs. At 7 day after egg laying, compared with controls (A), Ras^V12^/*lgl* ^−/−^‐induced tumour growth and invasion to the VNC (B) were suppressed by depleting *Toll*‐*7* (C and D) or expressing Puc (E). Expression of *Toll*‐*7*‐*IR* alone shows no obvious phenotype (F and G). Statistical analysis of the invasion percentage is shown in figures A’‐G’ (A’, 0%, *n =* 20; B’, 69.23%, *n =* 26; C’, 29.17%, *n =* 24; D’, 26.09%, *n =* 23; E’, 0%, *n =* 14; F’, 0%, *n =* 23; G’, 0%, *n =* 24). Fluorescent image of *Drosophila* 3rd instar larval wing imaginal discs (H–N) stained with anti‐MMP1 antibody (H’–N’) are shown. Anterior is to the left and dorsal up, *ptc*‐expressing cells were marked with GFP expression. Compared with controls (H–H’), *scrib* depletion‐induced cell migration (I) and MMP1 upregulation (I’) were suppressed by knocking‐down *Toll*‐*7* or expressing Puc (J–J’, K–K’ and L–L’). Expression of *Toll*‐*7*‐*IR* alone shows no obvious phenotype (M and N). (O) Quantification of tissue sizes ratio in figures A–G (*n =* 8; *n =* 13; *n =* 8; *n =* 9; *n =* 10; *n =* 11; *n =* 10), (P) Quantification of migrating cell numbers in H–N (*n =* 12; *n =* 9; *n =* 8; *n =* 8; *n =* 13; *n =* 10; *n =* 8) and (Q) Quantification of MMP1 positive cell numbers in H’–N’ (*n =* 12; *n =* 9; *n =* 8; *n =* 11; *n =* 17; *n =* 10; *n =* 8) are shown. One‐way ANOVA was used to compute *p*‐values, ****p < 0.0001, ***p < 0.001, n.s indicates not significant. Scale bar: 200 μm in A–G, 50 μm in H–N

It was shown previously that oncogenic cooperation of activated Ras (Ras^V12^) and *lgl* mutation (*lgl* ^−/−^) in *Drosophila* eye‐antennal discs could induce massive tumour‐like overgrowth and invasive metastasis to the ventral nerve cord (VNC).^13^, ^42^ Using this in‐vivo tumour model, we have been performing a genetic screen for modulators of tumour growth and invasion.^26^, ^43^ We found knockdown of *Toll*‐*7* by two independent *RNAi* lines significantly inhibited Ras^V12^/*lgl* ^−/−^‐triggered tumour growth in the eye‐antennal discs (Figure 1A–D, O) and reduced tumour invasion rate to the VNC from 69% to 29% and 26%, respectively (Figure 1A’–D’). Consistent with previous report that both tumour growth and invasion depends on the JNK signalling,^17^ expression of the JNK phosphatase Puckered (Puc), included as a positive control, blocked Ras^V12^/*lgl* ^−/−^‐induced tumour growth and invasive metastasis (Figure 1E, E’, O), while knockdown of *Toll*‐*7* alone showed no obvious effect (Figure 1F, G, F’, G’). Collectively, these results indicate that *Toll*‐*7* is necessary for Ras^V12^/*lgl* ^−/−^‐induced tumour‐like overgrowth and invasion.

To investigate the underlying mechanism by which depletion of *Toll*‐*7* suppressed Ras^V12^/*lgl* ^−/−^‐induced tumour growth, we checked cell death and cell proliferation by anti‐cDcp‐1 and anti‐phospho‐Histone H3 (pH3) staining, respectively. Knockdown of *Toll*‐*7* significantly suppressed Ras^V12^/*lgl* ^−/−^‐triggered cell proliferation (Figure S1A–A’, B–B’, C–C’), but did not increase cell death (Figure S1D–D’, E–E’, F–F’), suggesting that *Toll*‐*7* regulates cell proliferation, but not cell death, in the Ras^V12^/*lgl* ^−/−^ tumour model. A quantitative reverse transcription polymerase chain reaction (RT‐qPCR) assay was performed to check the knockdown efficiencies of the *Toll*‐*7 RNAi* lines (Figure S2).

### 3.2 Toll‐7 is required for cell polarity disruption‐induced invasive cell migration

Since the above tumour invasion phenotype also depends on early cell proliferation and tumorous growth, and *Toll*‐*7* depletion dramatically impedes Ras/*lgl* ^−/−^‐induced tumour overgrowth, the role of Toll‐7 in tumour invasion needs further verification. To this end, we employed another well‐established cell invasion model. In *Drosophila* wing discs, knockdown cell polarity gene *scrib* along the A/P compartment boundary driven by *ptc*‐Gal4 induces invasive cell migration with no obvious overgrowth phenotype.^28^ We counted the total number of migrating cells to quantify this phenotype and found that knockdown of *Toll*‐*7* resulted in decreased migrating cell number, while expression of Puc served as a positive control (Figure 1H–L, P).^44^ Meanwhile, loss‐of‐*scrib* also induced strong MMP1 expression, which is a biomarker for epithelial‐mesenchymal transition (EMT).^26^, ^28^, ^45^ Consistently, knockdown of *Toll*‐*7* suppressed *scrib* depletion‐induced MMP1 expression, while expression of Puc served as a positive control (Figure 1H’–L’, Q). Again, knockdown of *Toll*‐*7* alone did not cause any obvious phenotype (Figure 1M, N, M’, N’). Together, these results indicate that *Toll*‐*7* is required for disrupted cell polarity‐induced invasive cell migration.

### 3.3 Overexpression of Toll‐7 promotes tissue overgrowth and invasive migration

**FIGURE 2:**
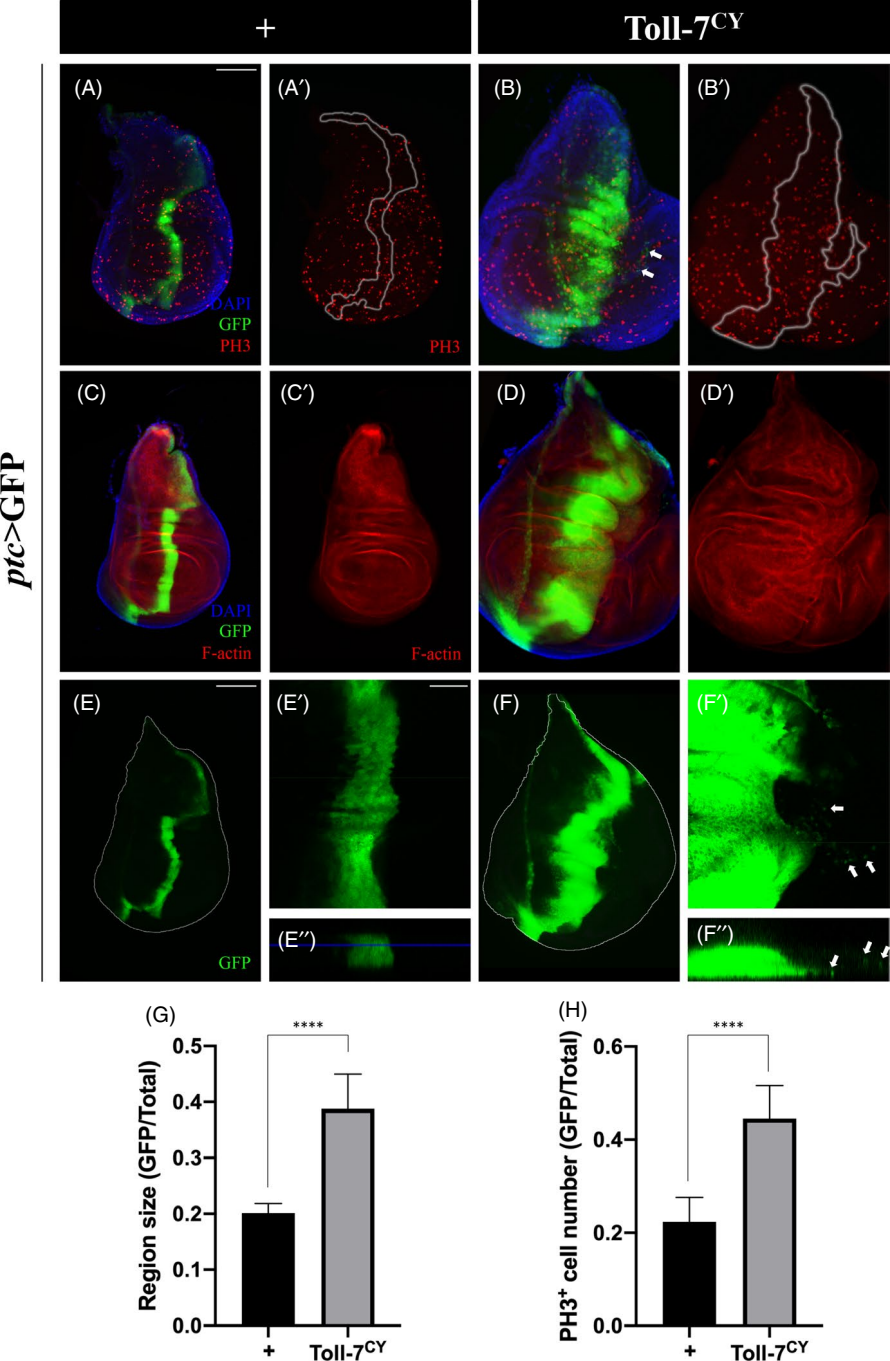
Ectopic Toll‐7 promotes cell proliferation and invasive migration. Fluorescent images of 3rd instar larval wing imaginal discs stained with anti‐PH3 antibody (A–B) or Phalloidin (C–D) are shown. Compared with the *ptc*‐Gal4 controls (A, C and E), overexpression of Toll‐7^CY^ results in enlarged GFP region (B, D and F), increased cell proliferation (B’), accumulated F‐actin (D’) and invasive cell migration (arrows in B, F’ and F”). E’ and F’ are high magnification views of E and F, while E” and F” are Z‐axis views of E’ and F’. (G and H) Statistical analysis of GFP region size/total size ratio in A and B (G, *n =* 10 for each group) and PH3‐postive cell number in A’ and B’ (H) are shown. The *t*‐test was used to compute *p*‐values, ****p < 0.0001. Scale bar: 100 μm in A–F, 50 μm in E’–F’

To test if Toll‐7 is sufficient to induce tissue overgrowth and invasion, we expressed Toll‐7^CY^, a constitutively active form of Toll‐7,^46^ along the A/P boundary in the wing discs. Compared with the *ptc*>GFP control, expression of Toll‐7^CY^ caused dramatic expansion of the GFP‐positive stripe (Figure 2A, B, G), accompanied by increased phospho‐Histone 3 (pH3) staining, a marker for mitosis,^47^ in the corresponding area (Figure 2A’, B’, H), suggesting Toll‐7 is sufficient to promote cell proliferation and tissue overgrowth. Meanwhile, we found some GFP‐positive cells are migrating away from the A/P boundary (arrows in Figure 2B), suggesting Toll‐7 is also sufficient to promote invasive cell migration. Consistently, Toll‐7^CY^ overexpression results in F‐actin accumulation (Figure 2C–C’, D–D’), E‐cadherin reduction (Figure S3A–A’, B–B’) and β‐integrin elevation (Figure S3C–C’, D–D’), which are well‐known EMT markers.^10^, ^48^, ^49^ Furthermore, we checked the Z‐section of the wing discs and found that Toll‐7^CY^ expressing cells were basally extruded and migrated from the A/P boundary (Figure 2E–E’’, F–F’’), which has been characterized as a typical cell invasion phenotype.^12^ Collectively, these results indicate that activated Toll‐7 is sufficient to promote tissue overgrowth and invasive cell migration.

### 3.4 Toll‐7 promotes cell proliferation and migration through Egr‐JNK signalling

**FIGURE 3:**
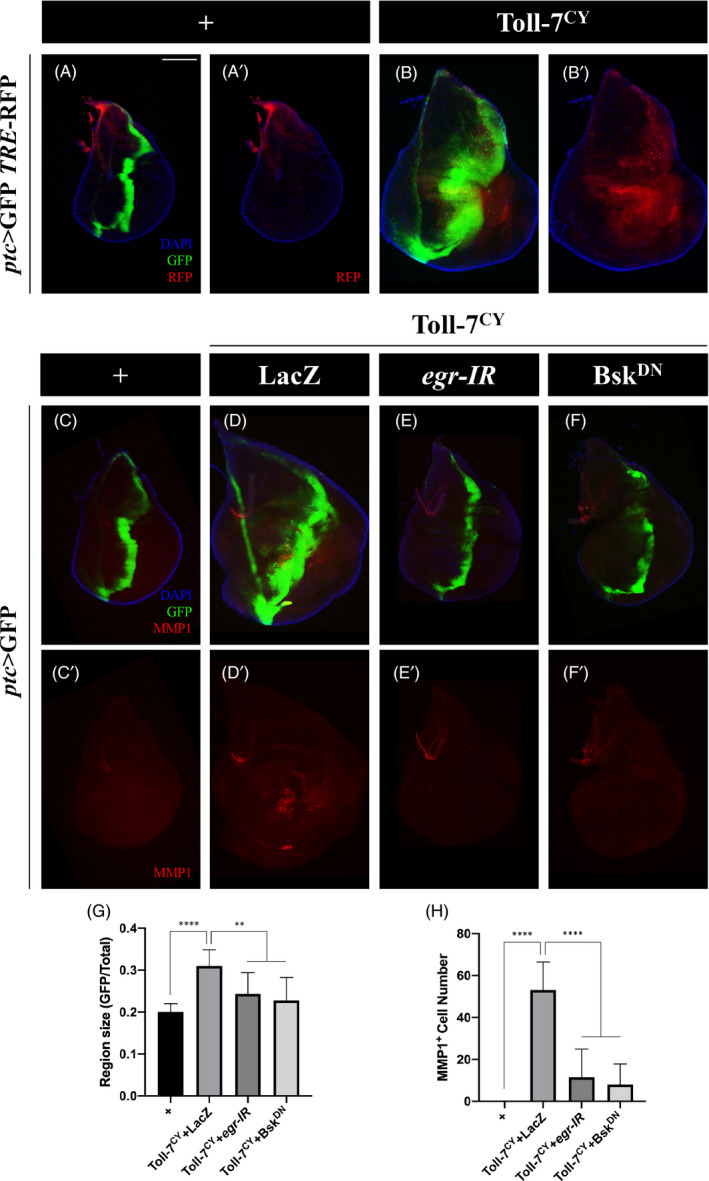
JNK signalling is necessary for Toll‐7‐ induced overgrowth and invasion. (A–F) Fluorescent images of 3rd instar larval wing imaginal discs stained with anti‐MMP1 antibody (C–F) are shown. Compared with the *ptc*‐Gal4 controls (A and C), ectopic Toll‐7 activates the JNK reporter *TRE*‐RFP (B), and induces cell invasion (D), which is suppressed by expressing *egr*‐*IR* (E) or Bsk^DN^ (F). (G and H) Statistical analysis of GFP region size/total size ratio in C–F (G, *n =* 12; *n =* 10; *n =* 11; *n =* 11) and MMP1 positive cell number in C’–F’ (H) are shown. One‐way ANOVA was used to compute *p*‐values, **p < 0.01, ****p < 0.0001. Scale bar: 100 μm in A–F

The Egr‐JNK signalling has been implicated in cell proliferation and invasion in fly tumour models.^17^, ^50^ To investigate the role of Egr‐JNK signalling in Toll‐7‐induced tissue overgrowth and invasion, we first checked whether JNK signalling is activated by ectopic Toll‐7. To this end, we examined the expression of two well‐known JNK pathway reporters, *TRE*‐RFP^51^ and *puc*‐lacZ,^52^ and found both of them were upregulated cell‐autonomously and non‐cell‐autonomously by Toll‐7^CY^ overexpression (Figure 3A–A’, B–B’ and Figure S4A–A’, B–B’). To directly assess the activation of JNK, we examined JNK phosphorylation by anti‐p‐JNK antibody staining and obtained similar results (Figure S4C–C’, D–D’). These data suggest that ectopic Toll‐7 suffices to activate JNK signalling.

Next, to investigate whether Egr‐JNK signalling is required for Toll‐7‐induced tissue growth and invasive cell migration, we blocked this signalling by depleting *egr* or expressing a dominant negative form of Basket (Bsk^DN^), the fly orthologue of JNK. Blocking Egr‐JNK signalling significantly impeded Toll‐7^CY^‐induced overgrowth and migration phenotypes (Figure 3C–F, G), as well as elevated MMP1 expression (Figure 3C’–F’, H), an EMT marker and also a JNK signalling reporter.^23^ These results indicate that Egr‐JNK signalling plays a critical role in Toll‐7‐induced tissue overgrowth and invasion and that Toll‐7 acts upstream of or in parallel to Egr.

### 3.5 Toll‐7 regulates Egr‐induced cell migration and JNK activation

**FIGURE 4:**
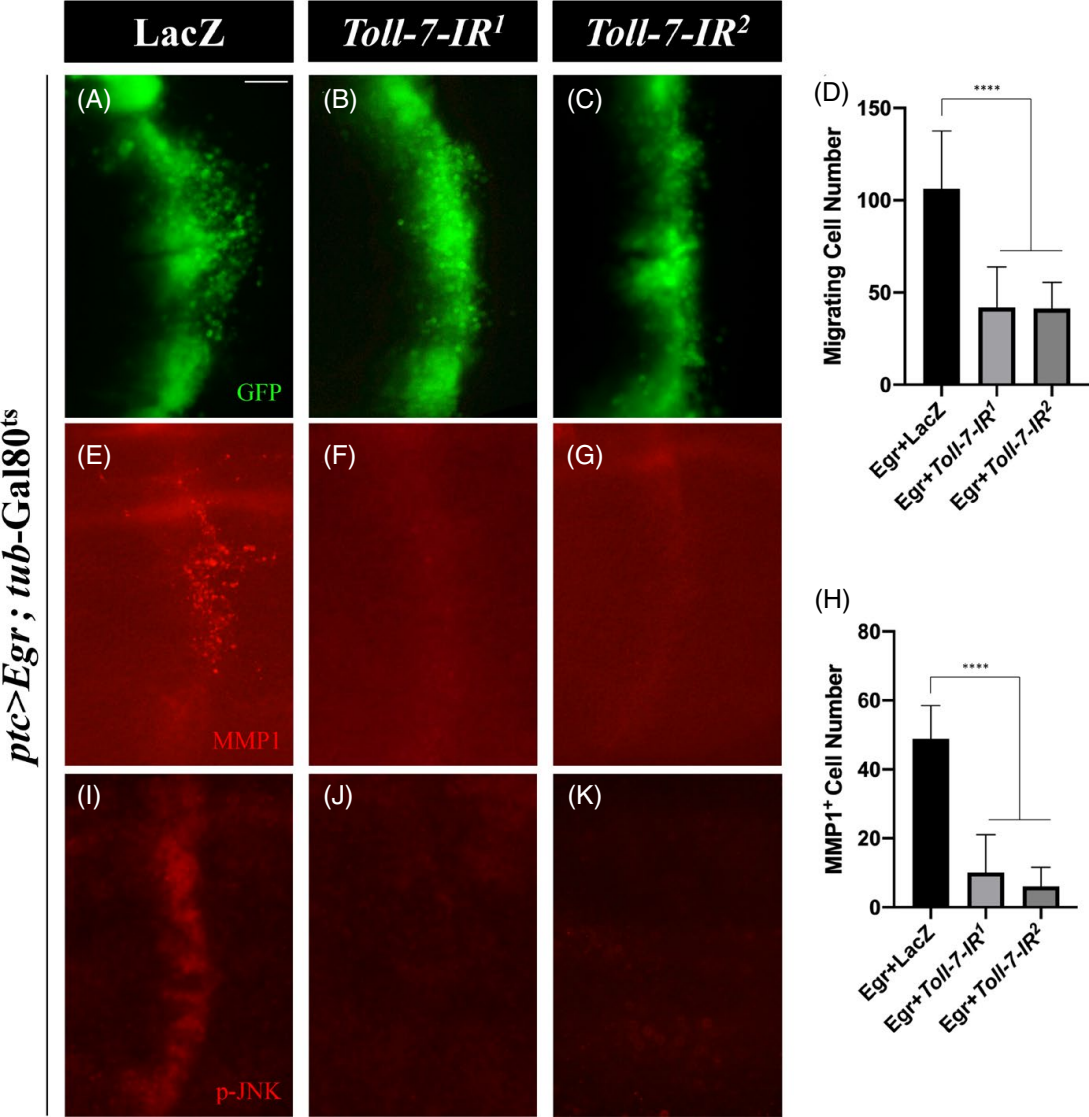
Loss of *Toll*‐*7* suppresses Egr‐induced cell migration and JNK activation. Fluorescent images of 3rd instar larval wing imaginal discs (A–C) stained with anti‐MMP1 (E–G) or anti‐p‐JNK antibody (I–K) are shown. Ectopic Egr‐triggered cell invasion (A), MMP1 (E) and p‐JNK (I) upregulation are suppressed by depletion of *Toll*‐*7* (B, C, F, G, J and K). (D and H) Quantification of migrating cell number in A–C (D, *n =* 9; *n =* 10; *n =* 10) and MMP1 positive cell number in E–G (H, *n =* 10; *n =* 8; *n =* 9) are shown. One‐way ANOVA was used to compute *p*‐values, ****p < 0.0001. Scale bar: 50 μm in A–C, E–G, I–K

The JNK signalling was reported to be both necessary and sufficient for cell migration in *Drosophila*.^26^, ^53^, ^54^ Overexpression of Egr driven by *ptc*‐Gal4 induced invasive cell migration and MMP1 upregulation.^28^ Intriguingly, both phenotypes were considerably suppressed by *Toll*‐*7* depletion (Figure 4A–H). Egr is known to activate JNK signalling and induce JNK‐dependent cell migration. We found that *ptc* > Egr‐induced JNK activation, detected by p‐JNK antibody, was also suppressed by depleting *Toll*‐*7* (Figure 4I–K). Together, these results indicate that Toll‐7 regulates Egr‐induced JNK activation and invasive cell migration.

### 3.6 Toll‐7 is required for endocytosis‐mediated early endosomal localization of Egr

**FIGURE 5:**
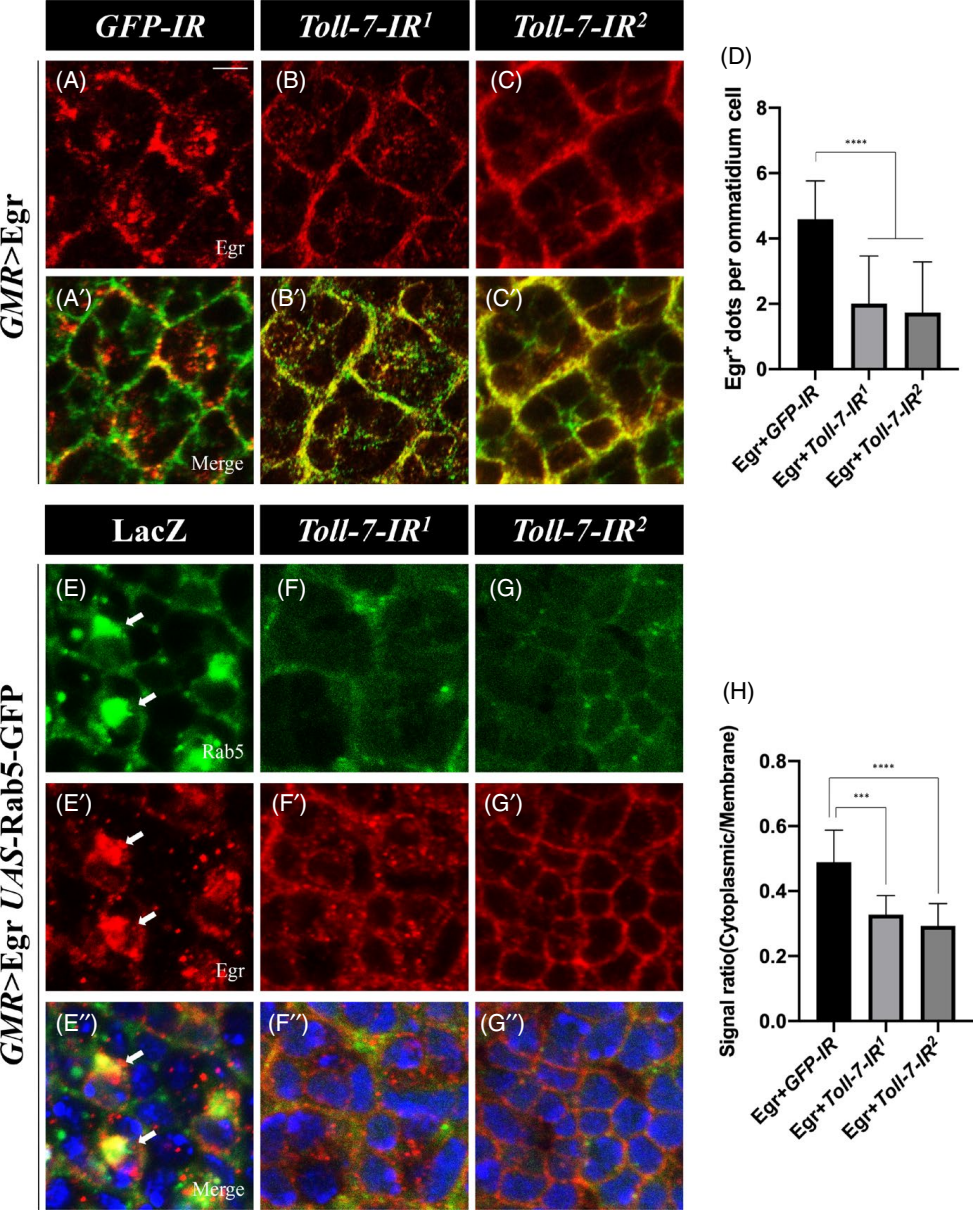
Toll‐7 is required for Egr cytoplasmic localization. (A–C) Fluorescent images of 3rd instar larval eye imaginal discs stained with anti‐Egr and anti‐Dlg antibodies are shown. Egr is mostly co‐localized with Dlg on the plasma membrane, with some punctate dots appeared in the cytoplasm (A). Egr cytoplasmic distribution is decreased by depletion of *Toll*‐*7* (B and C). (E–G) Fluorescent images of eye imaginal discs stained with anti‐Egr antibody are shown. Co‐localization of Egr and Rab5‐GFP in the early endosomes (arrows, E–E’’) is impeded by *Toll*‐*7* depletion (F and G). (D and H) Quantification of the numbers of cytoplasmic Eiger‐positive dots per ommatidium cell (D) and signal ratio of cytoplasmic/membrane Eiger (H) are shown. Scale bar: 10 μm in A–C, E–G

The above genetic data imply that Toll‐7 regulates JNK signalling in parallel to Egr. Previous study suggests that Egr is translocated from plasma membrane to endosomes through endocytosis and activates JNK signalling in the early endosomes.^55^ In addition, endocytosis is required for activation of Toll signalling and generation of the NF‐kB gradient during *Drosophila* embryogenesis.^56^ Based on these observations, we proposed that Toll‐7 might regulate JNK signalling via influencing Egr endocytosis. To test this hypothesis, we first checked the subcellular localization of Egr. Using Dlg antibody to mark the plasma membrane, we found Egr (red) was mainly co‐localized with Dlg (green) on the plasma membrane, while some punctate dots of Egr (red only) also appeared in the cytoplasm (Figure 5A–A’). Loss of *Toll*‐*7* decreased the cytoplasmic distribution of Egr (Figure 5B–B’, C–C’, D). Cytoplasmic Egr is mostly localized in the early endosomes,^55^, ^57^ which were visualized by Rab5‐GFP or anti‐Rab5 antibody, an early endosomal marker. Consistent with previous reports, expression of Rab5‐GFP not only labels the early endosomes, but also promotes endocytosis that results in increased proportion of Egr in the cytoplasm, mostly co‐localized with Rab5 in the early endosomes (arrows in Figure 5E–E”). Depletion of *Toll*‐*7* significantly blocked endocytosis and impeded cytoplasmic localization of Egr (Figure 5F–F’’, G–G’’, H and Figure S5A–A’’’, B–B’’’, C–C’’’). These results suggest that Toll‐7 is required for endocytosis and the early endosomal localization of Egr.

### 3.7 Toll‐7 promotes tissue growth through Hippo‐Yki signalling

**FIGURE 6:**
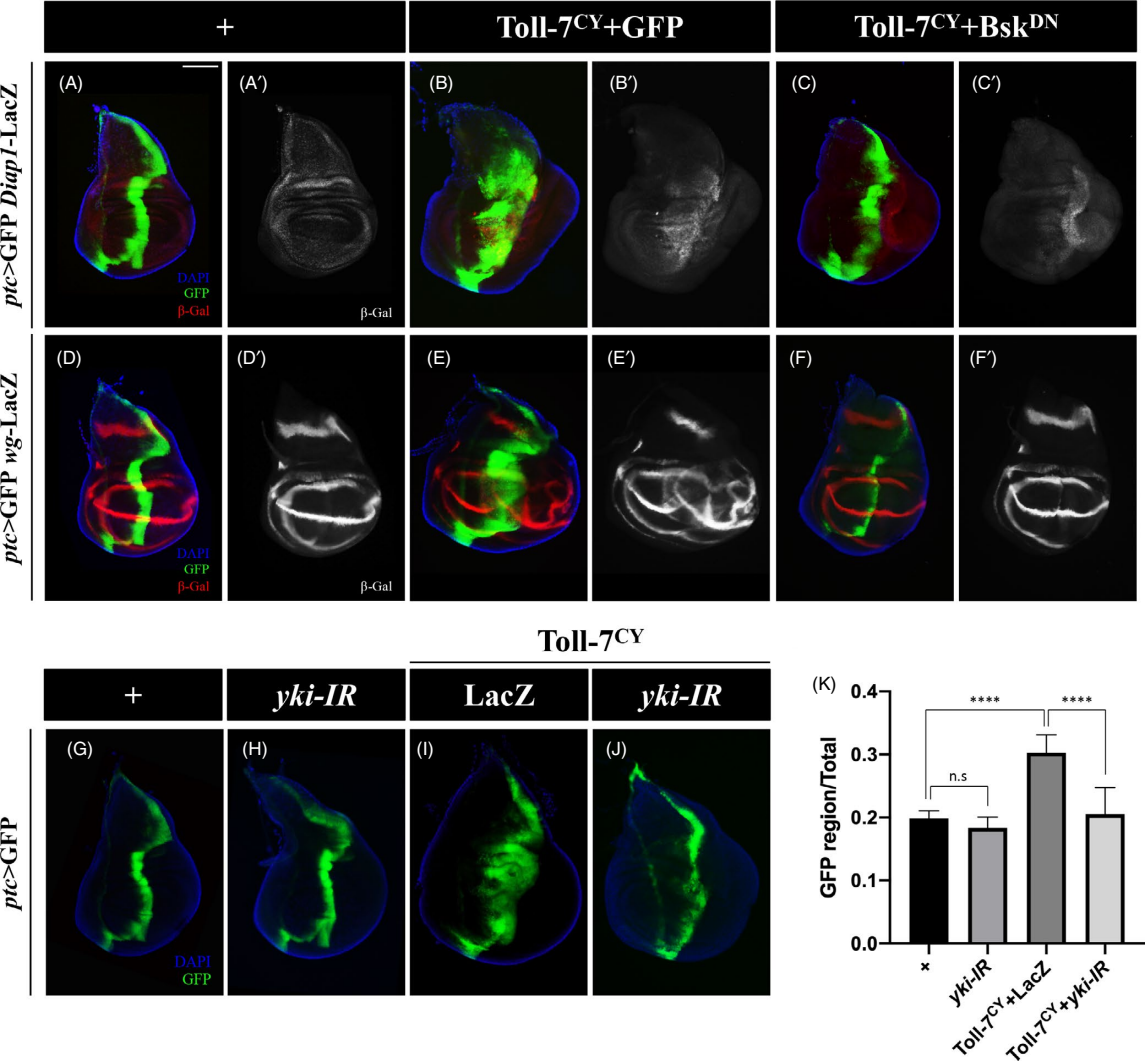
Hippo signalling is required for Toll‐7‐ induced overgrowth. (A–J) Fluorescent images of 3rd instar larval wing imaginal discs stained with anti‐β‐Gal antibody (A–F) are shown. Compared with the controls (A and D), ectopic Toll‐7 elevates Hippo pathway reporter *diap1*‐LacZ (B) and *wg*‐LacZ (E), which are partially suppressed by Bsk^DN^ (C and F). Meanwhile, compared with the *ptc*‐Gal4 control (G), ectopic Toll‐7‐induced overgrowth (I) is suppressed by *yki* knockdown (J), which by itself shows no obvious defects (H). (K) Quantification of G–J is shown. Scale bar: 100 μm in A–J

Although Toll‐7‐Egr‐JNK pathway could induce invasive cell migration, only Toll‐7, but not Egr‐JNK, suffices to promote cell proliferation and tissue overgrowth, suggesting this function of Toll‐7 depends on signalling(s) other than Egr‐JNK. The Hippo‐Yki signalling is a well‐known pathway that regulates cell proliferation and tissue growth.^58^ To investigate the relationship between Toll‐7 and Hippo signalling, we first checked the expression of Yki reporters, including *Diap1*‐LacZ, *wg*‐LacZ and Wg,^59^ and found they were all upregulated along the A/P boundary by *ptc* > Toll‐7^CY^ (Figure 6A–A’, B–B’, D–D’, E–E’ and Figure S6A–A’, B–B’). To further confirm this, we overexpressed Toll‐7^CY^ by *hh*‐Gal4 in the posterior compartment of wing discs and observed upregulation of *expanded* (Figure S7A–A’, B–B’) and *bantam* (Figure S7C–C’, D–D’), two additional Yki target genes.^60^ These data indicate that Toll‐7 is sufficient to induce Yki activation.

Previous studies reported that activated JNK signalling promotes Yki activation through inactivating Wats.^61^, ^62^ Consistently, blocking JNK signalling by expressing Bsk^DN^ partially suppressed Toll‐7^CY^‐ induced *Diap1*‐LacZ, *wg*‐LacZ and *wg* expression (Figure 6C–C’, F‐F’ and Figure S6D–D’), while *yki* depletion served as a positive control (Figure S6C–C’). Next, to investigate whether Toll‐7‐induced overgrowth depends on Hippo‐Yki pathway, we knocked down *yki* in the *ptc* > Toll‐7^CY^ background, and noted that Toll‐7‐induced overgrowth phenotype was suppressed by *yki* depletion (Figure 6G–K). These results indicate that Toll‐7 promotes tissue overgrowth through JNK‐dependent Yki activation.

### 3.8 Toll‐7 promotes EGFR signalling‐dependent tissue overgrowth

**FIGURE 7:**
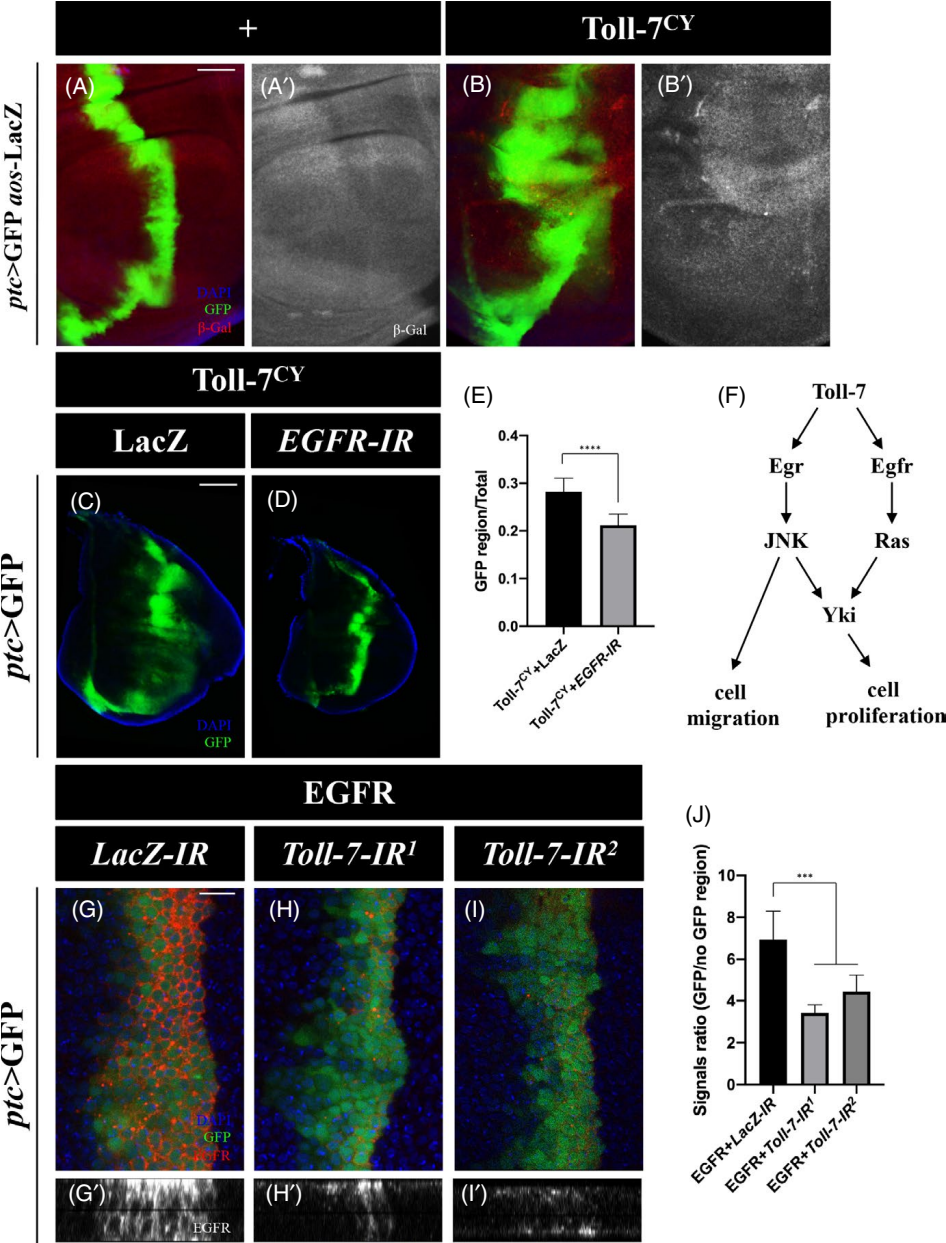
EGFR signalling is required for Toll‐7‐induced overgrowth. (A–D) Fluorescent images of 3rd instar larval wing imaginal discs stained with anti‐β‐Gal antibody (A and B). Compared with the *ptc*‐Gal4 control (A), Ectopic Toll‐7 activates EGFR signalling reporter *aos*‐LacZ (B). Ectopic Toll‐7‐induced overgrowth (C) is dramatically suppressed by knockdown of *EGFR* (D). (E) Statistical analysis of GFP region size/total size ratio in C (*n =* 11) and D (*n =* 8) is shown. (F) Model of Toll‐7‐induced cell proliferation and cell migration. (G–I) Fluorescent images of 3rd instar larval wing imaginal discs stained with anti‐EGFR antibody. Ectopic EGFR protein level driven by *ptc*‐Gal4 (G, Z‐axis view in G’) is diminished upon loss of *Toll*‐*7* (H and I, Z‐axis views in H’ and I’). (J) Quantification of EGFR signal ratio of GFP/no GFP region (*n =* 6 for each group). The one‐way ANOVA and T test were used to compute *p*‐values, ***p < 0.001, ****p < 0.0001. Scale bar: 100 μm in C–D, 50 μm in A–B, G–I

c‐Jun N‐terminal kinase signalling executes either anti‐ or pro‐tumour activity through differential regulation of Hippo‐Yki pathway. Activated Ras converts JNK signalling from an inhibitor to an activator of Yki, which promotes cell proliferation and tissue overgrowth.^63^, ^64^ Therefore, we proposed that Toll‐7 might activate two parallel pathways, Egr‐JNK and EGFR‐Ras. While Egr‐JNK signalling is sufficient to induce cell migration, it needs to cooperate with EGFR‐Ras pathway to activate Yki‐mediated cell proliferation and tissue overgrowth (Figure 7F). In support of this hypothesis, *ptc*‐Gal4 driven Toll‐7^CY^ overexpression elevated the expression of *aos*‐LacZ (Figure 7A–A’, B–B’), a reporter of EGFR pathway,^65^ confirming that Toll‐7 induces activation of EGFR‐Ras signalling. Furthermore, depletion of *EGFR* suppressed Toll‐7‐induced overgrowth phenotype (Figure 7C–E), indicating EGFR is required for Toll‐7‐induced tumorous growth. Intriguingly, ectopic EGFR protein level, produced by *ptc* > EGFR, was decreased upon *Toll*‐*7* depletion (Figure 7G–J), while endogenous EGFR level was enhanced upon Toll‐7^CY^ overexpression (Figure S8). Together, these data suggest Toll‐7 regulates EGFR expression and promotes EGFR signalling‐dependent tissue growth.

## 4 DISCUSSION

The *Drosophila* Toll receptor plays important roles in embryonic dorsal‐ventral patterning and innate immune response. Recently, increasing evidences suggest that Toll family receptors participate in multiple cellular events, including cell competition, cell death and tumour invasion.^38^, ^40^ Previous study reported that Toll6 regulates organ‐specific tumour metastasis,^42^ and spn5, a negative regulator of Toll signalling, is required for tumour‐suppressive cell competition, while activation of Toll signalling transforms loser cells to become super‐competitors in cell competition.^61^ Toll‐7 has been reported to regulate axon and dendrite targeting,^66^ yet its roles in tissue homeostasis and tumour progression remain unknown. In this study, by using well‐established *Drosophila* tumour models, we identified Toll‐7 as a novel regulator of tumour growth and invasion. We found loss of *Toll*‐*7* suppressed Ras^V12^/*lgl* ^−/−^‐induced tumour growth and invasion, and cell polarity disruption‐induced invasive cell migration. More importantly, ectopic Toll‐7 was sufficient to promote tumorous growth and invasion.

c‐Jun N‐terminal kinase signalling has been implicated in a wide range of biological processes and has been associated with Toll signalling in apoptosis.^35^, ^40^ We found Toll‐7‐induced tumour growth and invasion depend on Egr‐JNK signalling. Meanwhile, Toll‐7 is also required for Egr‐induced JNK activation and cell migration, suggesting Toll‐7 regulates JNK signalling by targeting Egr. Egr was reported to undergo endocytosis, and it activates JNK signalling in the early endosomes.^55^ We found loss of *Toll*‐*7* impedes endocytosis and Egr accumulation in the early endosomes, suggesting Toll‐7 induces JNK signalling by promoting Egr endocytosis. Intriguingly, TLR7, the mammalian orthologue of Toll‐7, is required for endosome mature in the innate immune cells in responding to pathogens,^67^ implying the function of Toll‐7 in endocytosis is likely conserved from fly to human. Additional studies are needed to further confirm and characterize this novel function, and to explore the underlying mechanism by which Toll‐7 regulates endocytosis.

c‐Jun N‐terminal kinase signalling executes either pro‐ or anti‐tumour function in different cellular contexts, depending on EGFR‐Ras signalling‐mediated switch of Yki activity.^63^ Consistently, we found both EGFR signalling and Yki were activated by Toll‐7, and both were required for Toll‐7‐ induced tumour growth, which also depends on JNK. Based on the previous work and the current study, we propose that Toll‐7 triggers both Egr‐JNK and EGFR‐Ras pathways in parallel. Subsequently, elevated EGFR‐Ras signalling converts Egr‐JNK signalling to an activator of Yki, which promotes cell proliferation and tissue overgrowth. On the other hand, Toll‐7‐ induced Egr‐JNK signalling suffices to promote invasive cell migration (Figure 7F). Consistent with our findings, Toll‐7 has been reported to activate pERK, a marker of EGFR signalling.^47^ We also provided evidence that Toll‐7 is necessary and sufficient for maintaining proper EGFR expression, most likely at the post‐transcriptional level, since *ptc*‐Gal4‐induced EGFR (*ptc* > EGFR) expression was significantly reduced upon *Toll*‐*7* depletion (Figure 7G–J). It remains to be elucidated how Toll‐7 regulates EGFR expression. Since our data suggest Toll‐7 regulates endocytosis, and endocytosis is known to be involved in EGFR recycling,^65^ Toll‐7 might regulate EGFR protein recycling through endocytosis.

Malignant tumour development requires multiple factors and signal transduction pathways, which have not been fully unveiled. Identifying novel regulators of tumour progression provides potential drug targets for cancer prevention and therapy. Toll/TLR signal pathway, which has conserved functions in innate immunity, is also considered to play a role in cancer inflammation.^68^ TLR3, TLR4 and TLR7 are upregulated in certain types of tumour cells, and their expression patterns are associated with tumour progression,^69^ suggesting that the innate immunity‐related factors may function as tumour promotors rather than suppressor. Yet, there is no in‐vivo report to back this notion, let alone the mechanism by which Toll‐related genes promote tumorigenesis+. Therefore, our results provide the first in‐vivo evidence for the pro‐tumour function of Toll family receptors, which may shed light on the development of novel therapeutic strategies and clinical treatments of related cancers.

## CONFLICT OF INTEREST

The authors declare no competing interest.

## AUTHOR CONTRIBUTIONS

X.D., Z.L. W.L. and L.X. conceived and designed the experiments. X.D., Z.L. and W.L. performed the experiments and analysed the data, G. L. and L.X. supervised the study, X.D., Z.L. and L.X. wrote the manuscript. All authors approved the final manuscript.

## Supporting information

Figure S1‐S8

## Data Availability

The data that support the findings of this study are available from the corresponding author upon reasonable request.
