# Supplementary material for: Toll‐7 promotes tumour growth and invasion in Drosophila
Source: Cell Prolif. 2022 Jan 20;55(2):e13188. doi: 10.1111/cpr.13188 (PMC8828261; doi:10.1111/cpr.13188)
Supplement: Supplementary file 1 — Figure S1‐S8 [file CPR-55-e13188-s001.docx]

**Toll-7 promotes tumor growth and invasion in *Drosophila***

Xiang Ding^1,#^, Zhuojie Li^1,#^, Gufa Lin^2^, Wenzhe Li^1,*^ and Lei Xue^1, 3,*^

^1^ Institute of Intervention Vessel, Shanghai 10th People's Hospital, Shanghai Key Laboratory of Signaling and Disease Research, School of Life Science and Technology, Tongji University, Shanghai, China

^2^ Key Laboratory of Spine and Spinal Cord Injury Repair and Regeneration of Ministry of Education, Orthopaedic Department of Tongji Hospital, School of Life Sciences and Technology, Tongji University, Shanghai, China

^3^ Zhuhai Precision Medical Center, Zhuhai People's Hospital, Zhuhai Hospital Affiliated with Jinan University, Zhuhai, Guangdong, China

^#^ These authors contribute equally to this work

^*^Correspondence: [lei.xue@tongji.edu.cn](mailto:lei.xue@tongji.edu.cn), [lwz@tongji.edu.cn](mailto:lwz@tongji.edu.cn)

**Supplementary Information**

Supplementary Figures

Detailed Genotypes

**
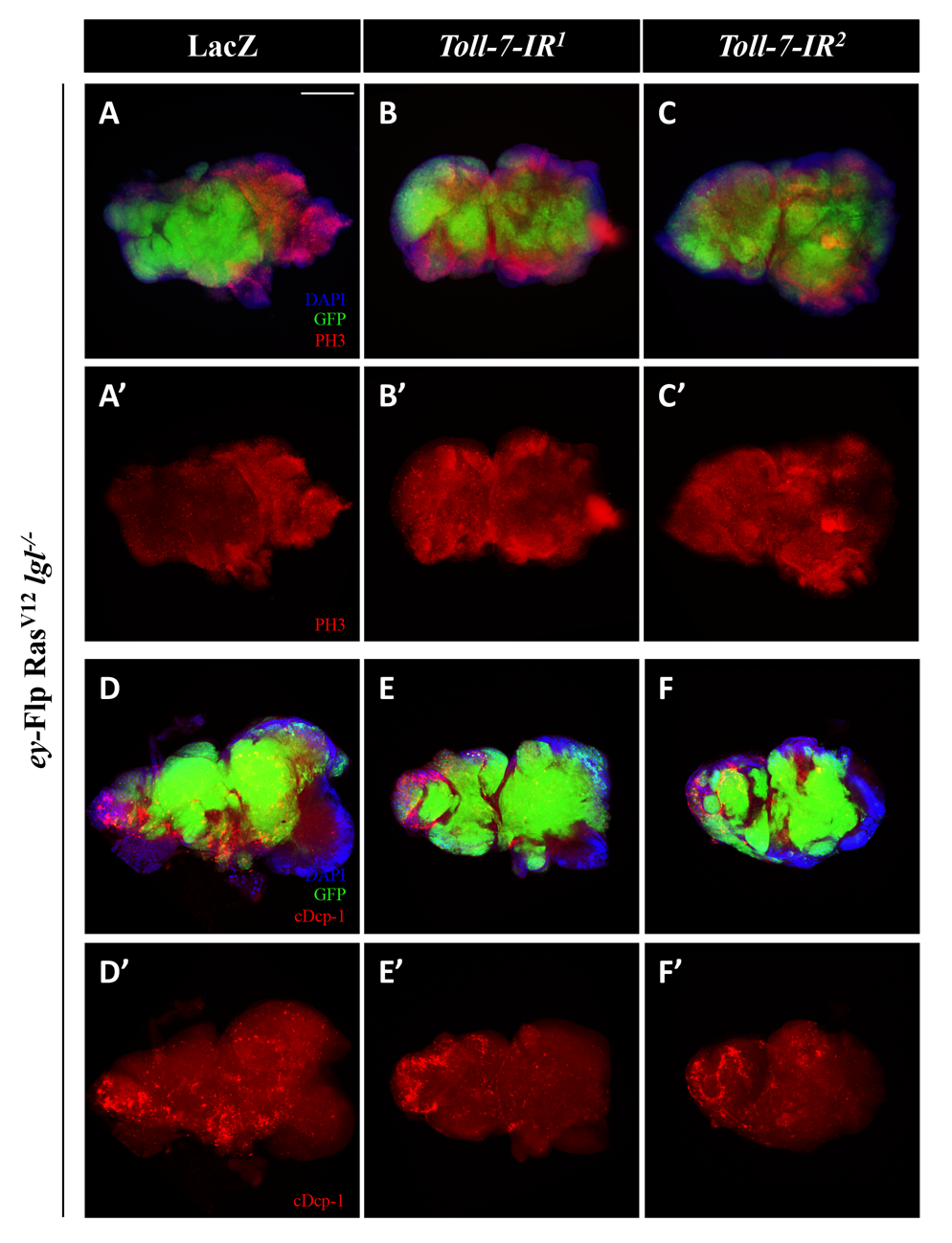
Supplement Figure 1**

**Figure S1. Depletion of *Toll-7* affects cell proliferation but not cell death.**

**(A**-**F)** Fluorescent images of *Drosophila* larval eye discs stained with anti-Phospho-Histone H3 **(A**-**C)** or anti-cDcp-1 **(D**-**F)** are shown (only part of the tumorous disc is shown in A). GFP-labeled MARCM clones were generated in the eye-antennal discs. Knockdown of *Toll-7* decreases cell proliferation **(A**-**C)**, but does not increase cell death **(D**-**F)** in Ras^V12^/*lgl*^-/-^ clones. Scale bar: 100 μm in **A**-**F**.

**
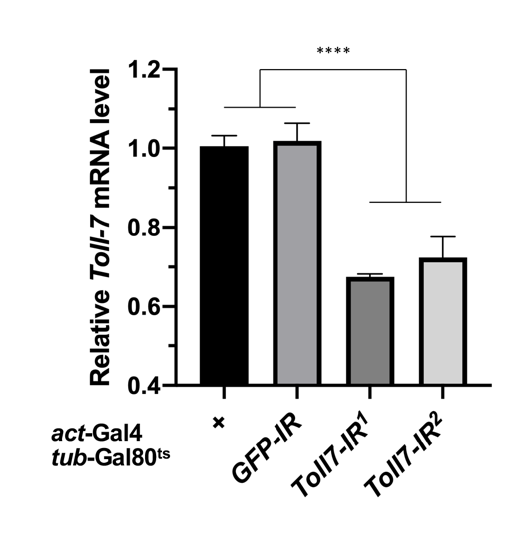
Supplement Figure 2**

**Figure S2. The knockdown efficacies of *Toll-7* RNAi lines.**

Expression of two independent *Toll-7* RNAi significantly reduces the level of *Toll-7* mRNA, as measured by quantitative RT-PCR. Error bars represents standard deviation from three independent experiments. One-way ANOVA with Bonferroni multiple comparison test was used to compute *P*-values, *****P*<0.0001.

**Supplement Figure 3
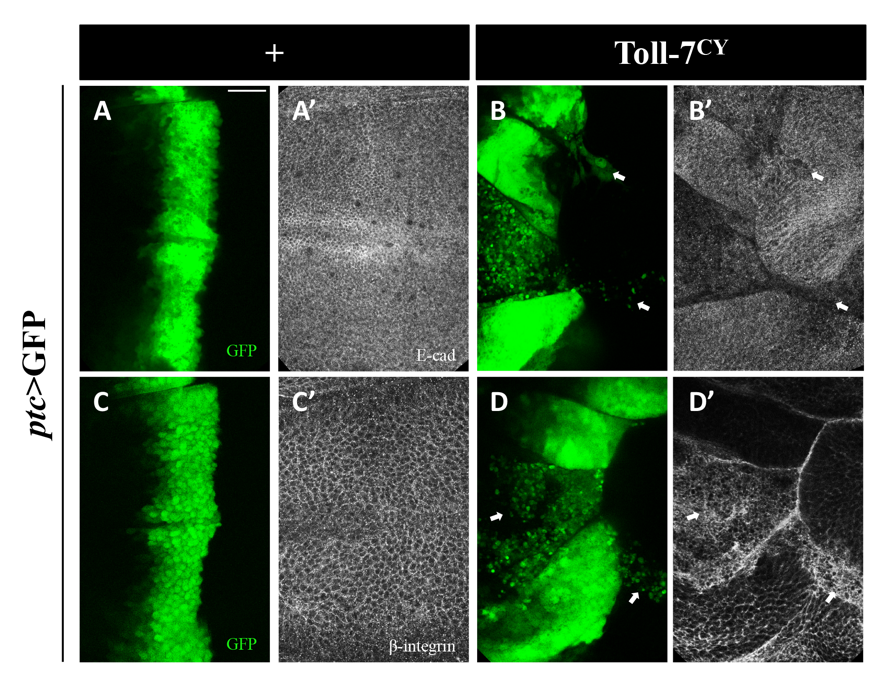
**

**Figure S3. Ectopic Toll-7 induces EMT-like cell migration.**

Fluorescent images of *Drosophila* larval wing discs stained with anti-E-cad (**A**-**B**) or anti-β-integrin (**C**-**D**) are shown. Compared with the controls (**A** and **C**), overexpression of Toll-7^CY^ results in cell migration (arrows), accompanied by reduced E-cad and enhanced β-integrin (**B** and **D**). Scale bar: 50μm in **A**-**D**.

**Supplement Figure 4**

**Figure S4. Ectopic Toll-7 activates JNK signaling**

Fluorescent images of 3^rd^ instar larval wing imaginal discs stained with anti-β-Gal (**A**-**B**) or anti-pJNK (**C**-**D**) antibody are shown. Compared with the controls (**A** and **C**), expression of Toll-7^CY^ driven by *ptc*-Gal4 activates *puc*-LacZ expression (**B**) and up-regulates p-JNK level (**D**). Scale bar: 100μm in **A**-**D**.


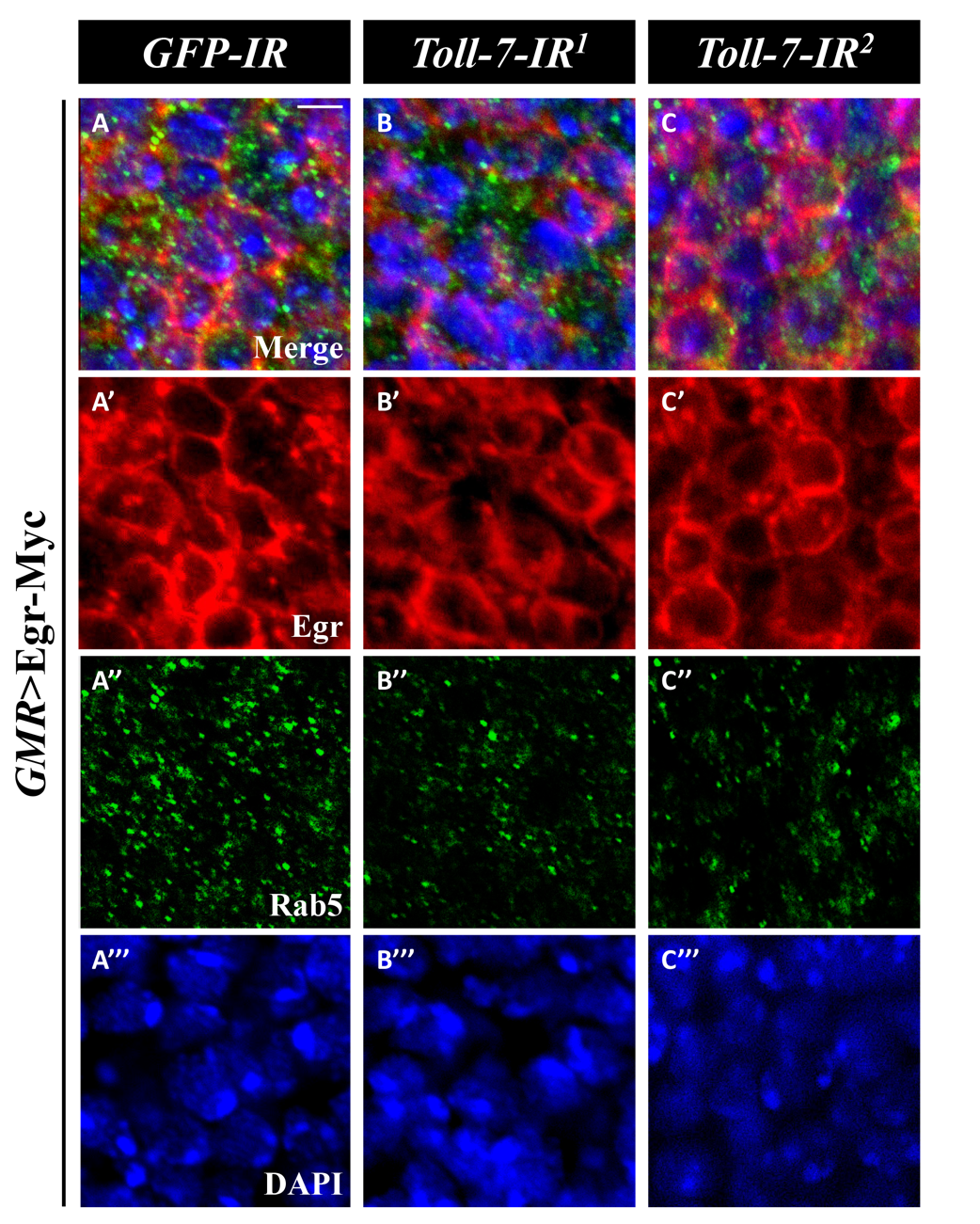
**Supplement Figure 5**

**Figure S5. Toll-7 is required for Egr localization in endosomes.**

**(A**-**C)** Fluorescent images of 3^rd^ instar larval eye imaginal discs co-stained with anti-Myc and anti-Rab5 antibodies are shown. The number of Rab5 dots as well as its co-localization with Egr **(A)** is significantly reduced upon knockdown of *Toll-7* **(B**, **C).** Scale bar: 10μm in **A**-**C**.

**Supplement Figure 6**

**Figure S6. Ectopic Toll-7 promotes JNK-dependent *wg* expression.**

(**A**-**D**) Fluorescent images of 3^rd^ instar larval wing imaginal discs stained with anti-Wg antibody are shown. Compared with the control (**A**), overexpression of Toll-7^CY^ elevates Wg expression (**B**), which is suppressed by expressing *yki-IR* (**C**) or Bsk^DN^ (**D**). Scale bar: 100μm in **A**-**D**.

**Supplement Figure 7**

**Figure S7. Ectopic Toll-7 activates Hippo target genes’ expression.**

(**A**-**D**) Fluorescent images of 3^rd^ instar larval wing imaginal discs stained with anti-β-Gal antibody. Expression of Toll-7^CY^ driven by *hh*-Gal4 up-regulates *ex*-LacZ (**B**) and *ban*-LacZ expression (**D**), compared with the controls (**A** and **C**). Scale bar: 100μm in **A**-**D**.

**Supplement Figure 8**

**Figure S8. Toll-7 promotes EGFR protein level.**

Fluorescent images of 3^rd^ instar larval wing imaginal discs stained with EGFR antibody. Expression of Toll-7^CY^ driven by *ptc*-Gal4 up-regulates EGFR protein level. Scale bar: 20μm.

**Genotypes in figures:**

**Figure 1**

**(A)** *yw ey*-Flp/+; *act*>y+>Gal4 *UAS*-GFP/*+*

**(B)** *yw ey*-Flp/+; *tub*-Gal80^ts^ FRT40A/*lgl^4^* FRT40A *UAS*-Ras^V12^; *act*>y+>Gal4 *UAS*-GFP/*UAS*-LacZ

**(C)** *yw ey*-Flp/+; *tub*-Gal80^ts^ FRT40A/*lgl^4^* FRT40A *UAS*-Ras^V12^; *act*>y+>Gal4 *UAS*-GFP/*UAS*-*Toll7-IR^BL30488^*

**(D)** *yw ey*-Flp/+; *tub*-Gal80^ts^ FRT40A/*lgl^4^* FRT40A *UAS*-Ras^V12^; *act*>y+>Gal4 *UAS*-GFP/*UAS*-*Toll7-IR^V39176^*

**(E)** *yw ey*-Flp/+; *tub*-Gal80^ts^ FRT40A/*lgl^4^* FRT40A *UAS*-Ras^V12^; *act*>y+>Gal4 *UAS*-GFP/*UAS*-Puc

**(F)** *yw ey*-Flp/+; *act*>y+>Gal4 *UAS*-GFP/*UAS*-*Toll7-IR^BL30488^*

**(G)** *yw ey*-Flp/+; *act*>y+>Gal4 *UAS*-GFP/*UAS*-*Toll7-IR^V39176^*

**(H)** *ptc*-Gal4 *UAS*-GFP/+

**(I)** *ptc*-Gal4 *UAS*-GFP/*UAS*-*scrib*-*IR*; *UAS-*LacZ/+

**(J)** *ptc*-Gal4 *UAS*-GFP/*UAS*-*scrib*-*IR*; *UAS*-*Toll7-IR^BL30488^*/+

**(K)** *ptc*-Gal4 *UAS*-GFP/*UAS*-*scrib*-*IR*; *UAS*-*Toll7-IR^V39176^*/+

**(L)** *ptc*-Gal4 *UAS*-GFP/*UAS*-*scrib*-*IR*; *UAS-*Puc/+

**(M)** *ptc*-Gal4 *UAS*-GFP/+; *UAS*-*Toll7-IR^BL30488^*/+

**(N)** *ptc*-Gal4 *UAS*-GFP/+; *UAS*-*Toll7-IR^V39176^*/+

**Figure 2**

**(A, C** and **E)** *ptc*-Gal4 *UAS*-GFP/+

**(B, D** and **F)** *ptc*-Gal4 *UAS*-GFP/+; *UAS*-Toll7^CY^/+

**Figure 3**

**(A)** *ptc*-Gal4 *UAS*-GFP *TRE*-RFP/+

**(B)** *ptc*-Gal4 *UAS*-GFP *TRE*-RFP/+; *UAS*-Toll7^CY^/+

**(C)** *ptc*-Gal4 *UAS*-GFP/+;

**(D)** *ptc*-Gal4 *UAS*-GFP/+; *UAS*-Toll7^CY^/*UAS*-LacZ

**(E)** *ptc*-Gal4 *UAS*-GFP/+; *UAS*-Toll7^CY^/*UAS*-*egr-IR*

**(F)** *ptc*-Gal4 *UAS*-GFP/+; *UAS*-Toll7^CY^/*UAS*-Bsk^DN^

**Figure 4**

**(A, E** and **I)** *ptc*-Gal4 *UAS*-GFP *UAS-Egr^Regg^*/+; *tub*-Gal80^ts^/*UAS*-LacZ

**(B, F** and **J)** *ptc*-Gal4 *UAS*-GFP *UAS-Egr^Regg^*/+; *tub*-Gal80^ts^/*UAS*-*Toll7-IR^BL30488^*

**(C, G** and **K)** *ptc*-Gal4 *UAS*-GFP *UAS-Egr^Regg^*/+; *tub*-Gal80^ts^/*UAS*-*Toll7-IR^V39176^*

**Figure 5**

**(A)** *UAS*-Egr^W^/+; *GMR-*Gal4^S^/*UAS-GFP-IR*

**(B)** *UAS*-Egr^W^/+; *GMR-*Gal4^S^/*UAS-Toll7-IR^BL30488^*

**(C)** *UAS*-Egr^W^/+; *GMR-*Gal4^S^/*UAS-Toll7-IR^V39176^*

**(E)** *UAS*-Egr^W^/*GMR-*Gal4; *UAS-*Rab5-GFP/*UAS*-LacZ

**(F)** *UAS*-Egr^W^/*GMR-*Gal4; *UAS-*Rab5-GFP/*UAS*-*Toll7-IR^BL30488^*

**(G)** *UAS*-Egr^W^/*GMR-*Gal4; *UAS-*Rab5-GFP/*UAS*-*Toll7-IR^V39176^*

**Figure 6**

**(A)** *ptc*-Gal4 *UAS*-GFP/+; *diap1-*LacZ/+

**(B)** *ptc*-Gal4 *UAS*-GFP/+; *UAS*-Toll7^CY^/*UAS*-GFP *diap1-*LacZ

**(C)** *ptc*-Gal4 *UAS*-GFP/+; *UAS*-Toll7^CY^/*UAS-*Bsk^DN^

**(D)** *ptc*-Gal4 *UAS*-GFP/*wg*-LacZ

**(E)** *ptc*-Gal4 *UAS*-GFP *wg*-LacZ/ +; *UAS*-Toll7^CY^/*UAS*-GFP

**(F)** *ptc*-Gal4 *UAS*-GFP *wg*-LacZ/ +; *UAS*-Toll7^CY^/*UAS-*Bsk^DN^

**(G)** *ptc*-Gal4 *UAS*-GFP/+;

**(H)** *ptc*-Gal4 *UAS*-GFP/+; *UAS*-*yki*-*IR*/+

**(I)** *ptc*-Gal4 *UAS*-GFP/+; *UAS*-Toll7^CY^/*UAS*-LacZ

**(J)** *ptc*-Gal4 *UAS*-GFP/+; *UAS*-Toll7^CY^/*UAS*-*yki-IR*

**Figure 7**

**(A)** *ptc*-Gal4 *UAS*-GFP/+; *aos*-LacZ/+

**(B)** *ptc*-Gal4 *UAS*-GFP/+; *UAS*-Toll7^CY^/*aos*-LacZ

**(C)** *ptc*-Gal4 *UAS*-GFP/+; *UAS*-Toll7^CY^/*UAS*-LacZ

**(D)** *ptc*-Gal4 *UAS*-GFP/+; *UAS*-Toll7^CY^/*UAS*-*EGFR-IR*

**(G)** *ptc*-Gal4 *UAS*-GFP/*UAS*-EGFR; *UAS-LacZ*-*IR*/+

**(H)** *ptc*-Gal4 *UAS*-GFP/*UAS*-EGFR; *UAS*-*Toll7-IR^BL30488^*/+

**(I)** *ptc*-Gal4 *UAS*-GFP/*UAS*-EGFR; *UAS*-*Toll7-IR^V39176^*/+

**Figure S1**

**(A** and **D)** *yw ey*-Flp/+; *tub*-Gal80^ts^ FRT40A/*lgl^4^* FRT40A *UAS*-Ras^V12^; *act*>y+>Gal4 *UAS*-GFP/*UAS*-LacZ

**(B** and **E)** *yw ey*-Flp/+; *tub*-Gal80^ts^ FRT40A/*lgl^4^* FRT40A *UAS*-Ras^V12^; *act*>y+>Gal4 *UAS*-GFP/*UAS*-*Toll-7-IR^BL30488^*

**(C** and **F)** *yw ey*-Flp/+; *tub*-Gal80^ts^ FRT40A/*lgl^4^* FRT40A *UAS*-Ras^V12^; *act*>y+>Gal4 *UAS*-GFP/*UAS*-*Toll-7-IR^V39176^*

**Figure S2**

From left to right:

1) *act*-Gal4/+; *tub*-Gal80^ts^/+

2) *act*-Gal4/+; *tub*-Gal80^ts^/*UAS-GFP-IR*

3) *act*-Gal4/+; *tub*-Gal80^ts^/*UAS*-*Toll-7-IR^BL30488^*

4) *act*-Gal4/+; *tub*-Gal80^ts^/*UAS*-*Toll-7-IR^V39176^*

**Figure S3**

**(A** and **C)** *ptc*-Gal4 *UAS*-GFP/+

**(B** and **D)** *ptc*-Gal4 *UAS*-GFP/+; *UAS*-Toll-7^CY^/+

**Figure S4**

**(A)** *ptc*-Gal4 *UAS*-GFP/+; *puc*-LacZ/+

**(B)** *ptc*-Gal4 *UAS*-GFP/+; *UAS*-Toll7^CY^/*puc*-LacZ

**(C)** *ptc*-Gal4 *UAS*-GFP/+

**(D)** *ptc*-Gal4 *UAS*-GFP/+; *UAS*-Toll7^CY^/+

**Figure S5**

**(A)** *UAS*-Myc-Egr-HA/+; *GMR*-Gal4/*UAS-GFP-IR*

**(B)** *UAS*-Myc-Egr-HA/+; *GMR*-Gal4/*UAS*-*Toll-7-IR^BL30488^*

**(C)** *UAS*-Myc-Egr-HA/+; *GMR*-Gal4/*UAS*-*Toll-7-IR^V39176^*

**Figure S6**

**(A)** *ptc*-Gal4 *UAS*-GFP/+;

**(B)** *ptc*-Gal4 *UAS*-GFP/+; *UAS*-Toll-7^CY^/*UAS*-LacZ

**(C)** *ptc*-Gal4 *UAS*-GFP/+; *UAS*-Toll-7^CY^/*UAS*-*yki-IR*

**(D)** *ptc*-Gal4 *UAS*-GFP/+; *UAS*-Toll-7^CY^/*UAS*-Bsk^DN^

**Figure S7**

**(A)** *ex*-LacZ/+; *hh-*Gal4 *UAS*-GFP/+

**(B)** *ex*-LacZ/+; *hh-*Gal4 *UAS*-GFP/*UAS*-Toll-7^CY^

**(C)** *ban*-LacZ/+; *hh-*Gal4 *UAS*-GFP/+

**(D)** *ban*-LacZ/+; *hh-*Gal4 *UAS*-GFP/*UAS*-Toll-7^CY^

**(E)** From left to right: 1) *ptc*-Gal4 *UAS*-GFP/+; 2) *ptc*-Gal4 *UAS*-GFP/+; *UAS-yki-IR/+*; 3) *ptc*-Gal4 *UAS*-GFP/+; *UAS-*Toll-7^CY^*/UAS*-LacZ; 4) *ptc*-Gal4 *UAS*-GFP/+; *UAS-*Toll-7^CY^*/UAS-yki-IR*

**Figure S8**

*ptc*-Gal4 *UAS*-GFP/+; *UAS-*Toll-7^CY^/+
